# Annotations

**Published:** 1895-02-02

**Authors:** 


### Annotations.

### "Flat" Men.

It is much easier to affirm that large numbers of one's fellow-men, including women, are "flat, stale, and unprofitable" in point of health, than to persuade them to pursue measures for their cure. Monotony, the wearisome monotony of town life and town occupations, is the chief factor in the production of this most undesirable state of things. A Masonic banquet, held at a large old-fashioned county inn, just beneath where the writer was sleeping on a particular night lately, reminded him that, in England, life was not always so dull and wearisome as it is now. Our forefathers, like the "Masons" in the Midland county town, used to feast royally, drink valiantly, and sing magnificently, at any rate so far as volume of voice was concerned. They tempered business and fortune-making with somewhat frequent outbursts of jollity, not to say drunkenness. Now we do not advise anybody to get drunk, far from it. But, if physiology may be permitted to say a word of sober truth, she will tell us that an occasional departure from routine in the way of food, and, let it be whispered low, sometimes enough of good wine to make one feel in good spirits, is of decided advantage to stomach, liver, and brain. It is as certain that the stomach, liver, and brain abhor too much routine in food and drink as it is that "nature abhors a vacuum," and indeed much more certain, for, according to modern physics, nature is much more indifferent to a vacuum than the older scientists used to believe. There is another factor in the case which is well worthy of the consideration of the strenuous man who means to make headway in these desperately competitive times. A change, a feast, a merry hour, unbends the bow, sweeps away the cerebral cobwebs, and brings up the gladiator fresh, smiling, and fit for the conflict. The gladiator in this condition can fight, and fight to some purpose. Your "flat man," on the other hand, lives a flat life, does flat things, and brings all his cattle to a flat market. Not thus was old England made, and not thus will modern England stand. We must have the old spirit back again, physiology tells us; and animated by that old spirit we shall be royal conquerors in all our individual and national battlefields, as our ancestors before us have been for many generations.

### The Memory of Lord Randolph Churchill.

The newspapers have discussed Lord Randolph Churchill's character and career with unwonted freedom, some of them hardly remembering that the saying, *de mortuis nil nisi bonum* is as worthy of being borne in mind to-day as it was on the day when it was first uttered. There is one fact of the first importance which, so far as we are aware, has been entirely, or almost entirely, overlooked by the newspapers. It is the fact that Lord Randolph's physical condition in all probability accounted for many circumstances in his life. The *Pall Mall Budget* publishes a portrait of Lord Randolph addressing the House of Commons, said to have been taken from life. By his side is sitting Mr. Balfour. The contrast between the two men is most striking. Mr. Balfour, who is really Lord Randolph's senior, looks placid, mentally healthy, and many years his junior. Lord Randolph looks old and haggard. Deep lines seam his face and forehead, and his eyes appear as if they were about to start from their sockets. The cause, we repeat, of all this is physical; and those who wish to do full justice to Lord Randolph Churchill's memory must insistently remember that a fatal disease had laid a relentless grip upon his brain and nervous system long before it finally broke him down and carried him to a premature grave.

### Drink Without Drunkenness.

In spite of persistent efforts to abolish it altogethe the public-house must long continue to exert a strong influence on the morals and health of the English people. American example has shown that attempted suppression defeats its own object, causing a wholesale evasion of the law, the effects of which are demoralising and disastrous. Local option, as an alternative measure, opens a vista of widespread confusion, and reformers are again turning to Norway and Sweden for inspiration, from whence excellent results from the adoption of the so-called Gothenburg system have from time to time been reported. Investigation of the working of the scheme brings out very clearly the fact that though drunkenness may be considerably lessened, and even practically abolished, the consumption of fermented beverages continues general. By the reasonable and practical, who do not wish to interfere with the liberty of the subject to the extent of complete prevention of all sale of intoxicants, the trial of such a system will be looked for with hope. Nor are we in England without an example of the results of such a system, tested by an experience of eighteen years. At Hampton Lucy, in Warwickshire, during this long period, the Rev. Osbert Mordaunt has conducted the business of the one public-house in the village. Mr. Mordaunt had to face one of two courses when entering into the charge of the parish, either to abolish the public-house altogether, and thus leave the parishioners to procure indifferent or bad drink at the nearest point, or through the grocer, or to personally try the experiment of supplying the community with good wholesome beer at a properly conducted establishment under the charge of a salaried manager, where the sale of intoxicants to those who had already had enough was prohibited. Mr. Mordaunt courageously and wisely took the latter course, in spite of the opposition and criticism he could not hope to escape when entering upon such an unusual clerical undertaking. Mr. Mordaunt showed further his excellent common-sense by making the business pay a reasonable amount, the profits being devoted to local charities and works, according to the Scandinavian system. The results have thoroughly justified the experiment. The inhabitants of Hampton Lucy obtain good and reasonably priced beer. Drunkenness is practically prevented, and the parish is benefited by the proceeds of the undertaking. Here, then, is an example of a reasonable and permanent success, undertaken and carried out in a courageous and wise manner by a clergyman of the Church of England, who has utilised a unique opportunity for the benefit of his parishioners. Besides the local good resulting from his action, Mr. Mordaunt showed the way to the Bishop of Chester and others by providing them with an object lesson, which could not fail to be instructive and helpful.

